# Associations between psoriatic arthritis and mental health among patients with psoriasis: A replication and extension study using the British Association of Dermatologists Biologics and Immunomodulators Register (BADBIR)

**DOI:** 10.1002/ski2.149

**Published:** 2022-07-06

**Authors:** Georgia Lada, Hector Chinoy, Peter S. Talbot, Richard B. Warren, C. Elise Kleyn, the BADBIR Study Group

**Affiliations:** ^1^ Dermatology Centre Salford Royal NHS Foundation Trust National Institute for Health Research Manchester Biomedical Research Centre The University of Manchester Manchester UK; ^2^ Division of Neuroscience and Experimental Psychology Faculty of Biology, Medicine and Health The University of Manchester Manchester UK; ^3^ National Institute for Health Research Manchester Biomedical Research Centre Manchester University NHS Foundation Trust The University of Manchester Manchester UK

## Abstract

**Background:**

Despite some evidence that psoriatic arthritis (PsA) may increase psychological burden in psoriasis, the mental health of this subpopulation is under‐investigated.

**Objectives:**

To investigate whether PsA is associated with higher depression and anxiety in moderate‐to‐severe psoriasis; explore whether pain mediates these associations; and estimate the prevalence of undiagnosed and untreated depression.

**Methods:**

Baseline data from British Association of Dermatologists Biologic and Immunomodulators Register (BADBIR) participants completing the Hospital Anxiety and Depression Scale (HADS) were analysed.

**Results:**

707 patients (*n* = 540 with psoriasis only; *n* = 167 with PsA) were included. Depression prevalence was higher in patients with than without PsA, when a HADS‐depression subscale cut‐off ≥8 was used (33% vs. 23%, adjusted Odds Ratio [OR] (95% Confidence Intervals [CI]) = 1.64 (1.09–2.45)), but did not differ using the HADS cut‐off ≥ 11. Anxiety prevalence was higher among PsA patients, regardless of HADS cut‐off (cut‐off ≥11: adjusted OR (95% CI) = 1.62 (1.07–2.45)). Pain fully mediated the effect of PsA on depression and anxiety in psoriasis. 53.6% of participants identified as depressed did not have a known psychiatric disorder; two thirds of depressed participants were not treated.

**Conclusions:**

PsA comorbidity in psoriasis is associated with higher anxiety; its association with depression appears to be robust when milder depressive syndromes are included, but less consistent for higher‐threshold depression definitions. Depression remains unrecognized and untreated in over half of moderately‐to‐severe psoriasis patients. Routine depression and anxiety screening is recommended in psoriasis and PsA. PsA comorbidity may increase depression and anxiety in psoriasis through pain experience.

## Graphical abstract

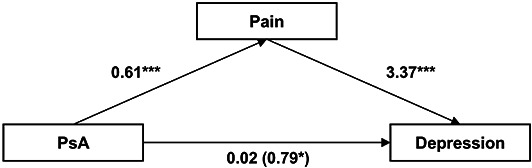

1

**What is already known about this topic?**

- Psoriasis patients with comorbid psoriatic arthritis (PsA) are a subpopulation with distinct clinical needs and putatively increased but under‐investigated psychological burden. It is unknown whether effects of PsA on patients' mental health can be attributed to pain experience.
- Depression is often underestimated in both dermatology and primary care settings. It is not clear how often depression remains undiagnosed in at‐risk populations with moderate‐to‐severe psoriasis.

**What does this study add?**

- The association of PsA with anxiety among psoriasis patients is robust and independent of other comorbidities. Its association with depression is replicated when milder depressive syndromes are included. Pain appears to fully mediate these associations.
- Depression remains unrecognized and untreated in over half of moderately‐to‐severe psoriasis patients.
- Routine depression and anxiety screening is recommended in psoriasis and PsA. For PsA, joint management of pain and mental health may prove beneficial.

## 2 INTRODUCTION

Depression and anxiety are overrepresented in psoriasis.^1^ Psoriatic arthritis (PsA) comorbidity may independently increase the psychological burden in psoriasis, however few studies have directly compared mental health in comorbid patients to the burden of psoriasis‐only populations.^1^, ^2^ Since depression and anxiety disorders are major contributors to disability and are associated with adverse outcomes in psoriasis,^3^ it is important to understand their relationships with psoriatic disease and develop prevention and management algorithms for high‐risk groups. A major bottleneck in depression management, even in populations at risk, is the fact that depression often remains undiagnosed; dermatologists may not recognise depression in over half of cases.^4^, ^5^

Depression prevalence and anxiety levels were higher in patients with PsA compared to patients with psoriasis only among participants we recruited from a single tertiary centre.^2^ However, in this previous sample, generalizability limitations due to population parameters or some effect size overestimation owing to the sample size (*n* = 219) cannot be excluded.

In the current study using the BADBIR database, we first tested the generalizability of our previous findings in a larger, multi‐centre sample, and investigated whether depression and anxiety are associated with PsA among patients with moderate‐to‐severe psoriasis. Second, we estimated the proportions of patients with moderate‐to‐severe psoriasis who were identified as being depressed but did not have a corresponding depression diagnosis or antidepressant treatment by any physician at the time of assessment. Third, we explored potential mediatory effects of pain in the relationship between PsA presence and affective (depression and anxiety) burden in psoriasis, given the significant overlap and the link between pain and depression in rheumatic disease.^6^

## 3 MATERIALS AND METHODS

### 3.1 Data source

BADBIR is a prospective cohort enrolling patients with moderate‐to‐severe psoriasis in over 160 sites in the United Kingdom and the Republic of Ireland. Its design has been published previously.^7^ BADBIR received ethics approval (reference 07/MRE08/9; see Supplementary Material S1).

Data until May 2020 were used in this study. We included all participants who completed the baseline Hospital Anxiety and Depression Scale (HADS)^8^; one participant missing information about PsA was excluded.

Baseline patient characteristics, including sociodemographic and clinical data, were obtained using a combination of patient interview, examination and screening of hospital records, performed by a trained professional. Information about PsA was recorded as response to the question: ‘Has the patient a diagnosis by a rheumatologist of psoriatic arthritis?’ and the year of disease onset was reported. At baseline, participants completed the HADS and the EuroQol (EQ‐5D‐3L).^9^

### 3.2 Outcome measures

Our primary outcome measure was the HADS,^8^ a validated screening tool for depression and anxiety in patients with physical illness. HADS was introduced as baseline assessment in BADBIR in 2019.

We report two definitions for depression and anxiety, using two thresholds (≥8 and ≥11) for each HADS subscale. A cut‐off ≥8 in the HADS‐Depression subscale (HADS‐D) shows optimal psychometric properties for identifying major depression (specificity/sensitivity: 0.82/0.74). A cut‐off ≥11 is more specific, but less sensitive (0.92/0.56).^10^ There is less evidence on the HADS‐anxiety subscale (HADS‐A); a meta‐analysis found sensitivity/specificity of 0.78/0.74 for identifying generalized anxiety disorder (GAD) using scores ≥8.^10^ Complementarily to these definitions, we report overall HADS‐subscale scores as indicators of depressive and anxiety symptom burden for comparative purposes. Three severity bands have been proposed for each HADS subscale (8–10: mild, 11–14: moderate, 15–21: severe depression/anxiety).^11^

To define an existing diagnosis of depression, we used the reported Medical Dictionary for Regulatory Activities (MedDRA) High Level Term ‘Depressive disorders’. We also examined the reported preferred terms for all psychiatric disorders in our sample to reduce misclassification bias.

### 3.3 Data analysis

We used chi‐square tests, *t*‐tests and Mann Whitney *U* tests where appropriate to compare baseline characteristics between the groups.

We investigated associations of PsA with depression and anxiety prevalence using logistic regression and controlling for age, gender, ethnicity, physical comorbidities (as having at least one of the following: cardiovascular disease, cancer, inflammatory bowel disease, endocrine, other musculoskeletal, severe systemic, central nervous system disease or severe chronic infections), psoriasis severity measured with the Psoriasis Area and Severity Index (PASI), and Body Mass Index (BMI).

We used *t*‐tests to investigate differences in mean HADS‐subscale scores between the groups; and linear regression models to control for confounders in the associations between HADS scores and PsA.

For our analysis, we excluded PASI scores according to a set of criteria, which took account of the temporal relationship of PASI to the HADS assessment and start of treatment. The criteria are described in the Supplement.

Due to high covariate missingness (33.38% of sample), we imputed missing data (PASI and BMI) using multiple imputation (MI) before adding them to the regression models; data were assumed to be missing at random.^12^ We corrected for multiple comparisons in all primary outcomes using the Benjamini‐Hochberg procedure to control for the false discovery rate (FDR).^13^

As a next step, we conducted an exploratory, statistical mediation analysis to investigate the role of pain in the association of PsA with depression and anxiety levels. For this, we hypothesized two mediational models. In both models, we used the presence of an existing PsA diagnosis as the independent variable and the presence of pain/discomfort as reported in the Euroqol 3‐level questionnaire item as a binary mediator variable (‘yes’ corresponding to having any pain or discomfort and ‘no’ corresponding to having no pain or discomfort). As outcome variable, the score in the HADS‐D depression subscale was used in the first (depression) model and the score in the HADS‐A anxiety subscale was used in the second (anxiety) model. We excluded *n* = 15 participants where the HADS assessment was obtained before the Euroqol.

Each hypothesized pathway was tested using nonparametric bootstrapping with 5000 iterations. We controlled for potential confounders, including age, gender, ethnicity (white/non‐white), and physical comorbidity as defined previously. Furthermore, we conducted a sensitivity analysis to investigate the degree to which our findings were robust to a potential violation of the sequential ignorability assumption, using the correlated residuals method.^14^

Finally, we investigated the representativeness of our sample within BADBIR using classic statistical tests. All statistical analysis was performed in *R*.^15^

## 4 RESULTS

### 4.1 Population characteristics

Seven hundred and seven BADBIR participants were included in the study; *n* = 540 with psoriasis only and *n* = 167 with both psoriasis and PsA. Baseline sociodemographic and psoriasis‐related clinical characteristics are reported in Table 1. Participants with PsA were older compared to participants without PsA; they did not differ for age at psoriasis onset, PASI or dermatology‐related quality of life (Table 1).

**TABLE 1:** Sociodemographic and disease characteristics

Baseline characteristics	*N*	Missing data (%)	All (*n* = 707)	Psoriasis only (*n* = 540)	PsA (*n* = 167)	*p*‐value
Age, mean (SD)	707	0 (0.0)	46.5 (13.0)	45.7 (13.0)	48.8 (12.6)	0.006
Gender (female)	707	0 (0.0)	305 (43.1%)	229 (42.4%)	76 (45.5%)	0.537
Work status (works or studies)	693	14 (1.9)	535 (77.2%)	417 (77.2%)	118 (70.7%)	0.251
Ethnicity (white)	707	0 (0.0)	630 (89.1%)	490 (90.7%)	140 (83.8%)	**0.029**
Psoriasis duration in years, median (IQR)	698	9 (1.3)	20.0 (18.7)	19.0 (19)	21.0 (18.2)	0.178
Age at psoriasis onset in years, median (IQR)	698	9 (1.3)	22.0 (18.0)	22.0 (18.0)	23.0 (19.2)	0.633
PASI score; any date,^a^ median (IQR)	705	2 (0.3)	10.7 (12.2)	10.5 (12.4)	10.7 (12.0)	0.760
PASI score; filtered date,^b^ median (IQR)	511	196 (27.7)	10.0 (12.3)	10.0 (12.2)	8.5 (11.5)	0.294
DLQI score; any date,^a^ median (IQR)	692	15 (2.1)	17 (13)	16 (12)	17 (13)	0.558
DLQI score; filtered date,^b^ median (IQR)	391	316 (53.2)	16 (13)	16 (13)	15.5 (16)	0.800
Cohort	707	0 (0.0)				
Biologics			700 (99.0%)	536 (99.3%)	164 (98.2%)	0.212
Conventional			3 (0.4%)	1 (0.2%)	2 (1.2%)	
Small molecule			4 (0.6%)	3 (0.6%)	1 (0.6%)	
Had ever tried biologics before	704	3 (0.4)	180 (15.5%)	116 (21.5%)	64 (38.8%)	**<0.001**
Experiencing pain/Discomfort (EQ5D‐3L)^c^	685	18(2.6)	367 (53.6%)	250 (47.5%)	117 (71.7%)	**<0.001** ^d^
Level 0 (no)			328 (47.9%)	282 (53.0%)	46 (28.2%)	
Level 1 (moderate)			305 (44.5%)	210 (39.5%)	95 (58.3%)	
Level 2 (extreme)			62 (9.0%)	40 (7.5%)	22 (13.5%)	

Abbreviations: DLQI, Dermatology Life Quality Index^31^; EQ5‐3L, EuroQol questionnaire; HADS, Hospital Anxiety and Depression Scale; IQR, interquartile range; PASI, Psoriasis Area and Severity Index; PsA, group with psoriatic arthritis; SD, standard deviation. *p*<0.05 are reported in bold.

^a^ Score dated closest to the HADS reported if more than one scores at baseline.

^b^ Filtered based on temporal criteria in relation to the HADS and drug initiation dates (described in the data analysis section).

^c^ Patients who responded having pain or discomfort; corresponds to levels 2 and 3 in the EQ5D‐3L pain/discomfort item.

^d^ Kruskal‐Wallis test for comparison between groups for all three EQ5D‐3L item levels also yielded a *p*‐value <0.001.

Lifestyle factors and psychiatric history of the cohort are presented in Table 2. The only reported term for depressive disorder in our sample was ‘depression’; there was no patient with bipolar, postpartum or other depressive disorder. Reported depression comorbidity and antidepressant use at baseline were 23% and 16% for patients with PsA and 20% and 14% for patients without PsA. Most patients using antidepressants were treated with SSRI (Selective Serotonin Reuptake Inhibitors) monotherapy. 25% of treated depressed patients were receiving antidepressant combinations or augmentation with lithium/antipsychotics, reflecting higher disease severity. The groups did not differ for the presence of depression, psychiatric history or antidepressant use. Prevalence of opioid analgesic use tended to be higher in the group with PsA, mirroring the higher levels of pain in this group (Table 2).

**TABLE 2:** Lifestyle characteristics and psychiatric history

Baseline characteristics	*N*	Missing data (%)	All (*n* = 707)	Psoriasis only (*n* = 540)	PsA (*n* = 167)	*p*‐value
BMI; median (IQR)	656	51 (7.2)	30.3 (9.1)	30.1 (8.3)	31.6 (9.5)	0.098
Current smoker	696	11 (1.5)	143 (20.5%)	117 (22%)	26 (16%)	0.112
Any physical comorbidities	707	0 (0.0)	419 (59.3%)	310 (57.4%)	109 (65.3%)	0.080
Psychiatric disorders (any)	707	0 (0.0)	166 (23.5%)	124 (23.0%)	42 (25.1%)	0.632
Diagnosed depression	707	0 (0.0)	147 (20.8%)	108 (20.0%)	39 (23.3%)	0.409
Antidepressants (AD)	707	0 (0.0)	102 (14.4%)	76 (14.1%)	26 (15.6%)	0.723
SSRIs			64 (62.7%)	51 (67.1%)	13 (50.0%)	0.186^a^
SNRIs			15 (14.7%)	11 (14.4%)	4 (15.3%)	
Mirtazapine			13 (12.7%)	7 (9.2%)	6 (23.1%)	
TCAs			26 (25.5%)	16 (21%)	10 (38.5%)	
AD combinations			13 (12.7%)	7 (13.7%)	6 (23.1%)	0.136
Opioid analgesics	707	0 (0.0)	51 (7.2%)	33 (6.1%)	18 (10.8%)	0.062

*Note*: Descriptive statistics are reported in non‐missing observations.

Abbreviations: BMI, Body Mass Index; IQR, interquartile range; PsA, group with psoriatic arthritis; SD, standard deviation; SNRIs, Serotonin‐norepinephrine reuptake Inhibitors; SSRIs, Selective Serotonin Reuptake Inhibitors; TCAs, Tricyclic antidepressants.

^a^ Versus all non‐SSRI antidepressants.

### 4.2 Depression and anxiety in patients with and without psoriatic arthritis

The prevalence of depression was higher in patients with PsA than in patients without PsA using a HADS cut‐off of 8: 32.9% versus 22.7%, Odds Ratio (OR) (95% Confidence Intervals [CI]) = 1.66 (1.13–2.43). The association between PsA and depression remained significant after adjusting for potential confounders. However, when we increased the HADS threshold to 11, PsA was no longer associated with depression.

Patients with PsA had a higher prevalence of anxiety compared to patients with psoriasis only, regardless of the HADS cut‐off used to define anxiety (for cut‐off ≥8: 44.3% vs. 35.2%, adjusted OR (95% CI) = 1.51 (1.04–2.19); for cut‐off ≥11: 28.7% versus 20.2%, adjusted OR (95% CI) = 1.62 (1.07–2.45)) (Table 3).

**TABLE 3:** Prevalence and Odds Ratio (OR) of depression and anxiety for presence of psoriatic arthritis (PsA) compared to patients with psoriasis only

Outcomes measured with HADS	Prevalence (count)			OR crude (95% CI)	*p*‐value	OR adjusted (95% CI)^a^	*p*‐value
	Psoriasis only	PsA	All				
Depression, cut off ≥8	22.7% (123)	32.9% (55)	25.2% (178)	1.66 (1.13–2.43)	**0.026** ^b^	1.64 (1.09–2.45)	**0.037** ^c^
Depression, cut off ≥11	11.5% (62)	11.9% (20)	11.6% (82)	1.05 (0.60–1.77)	0.862^d^	0.94 (0.52–1.63)	0.862^e^
Anxiety, cut off ≥8	35.2% (190)	44.3% (74)	37.3% (264)	1.47 (1.03–2.08)	**0.040** ^f^	1.51 (1.04–2.19)	**0.038** ^g^
Anxiety, cut off ≥11	20.2% (109)	28.7% (48)	22.2% (157)	1.59 (1.07–2.36)	**0.037** ^h^	1.62 (1.07–2.45)	**0.037** ^i^

Abbreviations: CI, confidence intervals; HADS, Hospital Anxiety and Depression Scale. *p*<0.05 are reported in bold.

^a^ Adjusted for: age, gender, ethnicity, presence of physical comorbidities, psoriasis severity and body mass index.

^b^ BH (Benjamini‐Hochberg)‐corrected; original *p*‐value = 0.009.

^c^ BH‐corrected; original *p*‐value = 0.017.

^d^ BH‐corrected; original *p*‐value = 0.862.

^e^ BH‐corrected; original *p*‐value = 0.661.

^f^ BH‐corrected; original *p*‐value = 0.034.

^g^ BH‐corrected; original *p*‐value = 0.029.

^h^ BH‐corrected; original *p*‐value = 0.021.

^i^ BH‐corrected; original *p*‐value = 0.022.

Comparing the two patient groups for total raw HADS‐subscale scores, scores for depression and anxiety were higher in the PsA group, even after adjustment for confounders (depression: β (95% CI) = 0.84 (0.10, 1.57), *p* = 0.037; anxiety: β (95% CI) = 1.32 (0.54, 2.11), *p* = 0.012, Table S1).

### 4.3 Mediating effects of pain

**FIGURE 1:**
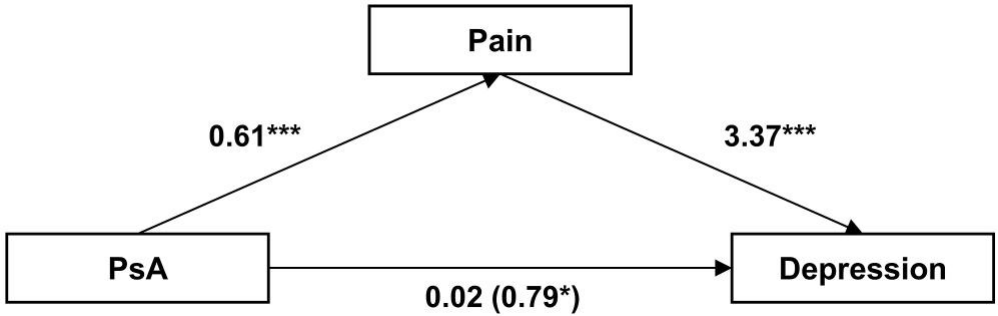
Mediation model. Indirect effect of psoriatic arthritis (PsA) on depression score at baseline (as measured by the Hospital Anxiety and Depression depression subscale HADS‐D) through pain. Model is controlled for age, gender, ethnicity and presence of other physical comorbidities. The effects on the direct path from PsA to depression show the direct effect (total effect). Direct effect was not statistically significant. *p < 0.05, ***p < 0.001

We aimed to explore the effect of experiencing pain (or discomfort) in the association between having a diagnosis of psoriatic arthritis and levels of depression at baseline. There were associations between PsA and pain, as well as pain and depression levels. As hypothesized, the effect of PsA on the HADS‐D score was fully mediated by the presence of pain, after controlling for age, gender, ethnicity and other physical comorbidities (Figure 1). The bootstrapped indirect effect was *β* = 0.77, 95% CI 0.42 *to* 1.13, *p* < 0.001. The proportion of effect mediated by pain was 0.98 (*average causal mediation effects [ACME]/total effect = *0.77/0.79; 95% CI 0.25–5.43).

Our sensitivity analysis found *R* ^2^ = 0.36 for residual variance and *R* ^2^ = 0.26 for total variance, indicating that an unmeasured confounder can account for up to 26% of the total variation before ACME becomes zero. A *ρ* value of 0.60 (*ρ* representing correlation between residuals) was needed to reverse the sign of ACME (and therefore invalidate the existence of a mediated effect of PsA on depression through pain). This suggests relatively low likelihood of the observed mediated effect to be caused by an unobserved confounder and robustness of our findings to the sequential ignorability assumption.

**FIGURE 2:**
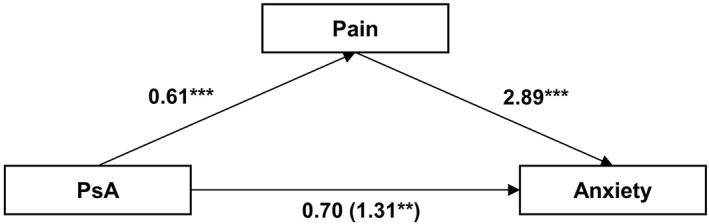
Mediation model. Indirect effect of psoriatic arthritis (PsA) on current anxiety score (as measured by the Hospital Anxiety and Depression anxiety subscale HADS‐A) through pain. Model is controlled for age, gender, ethnicity and presence of other physical comorbidities. The effects on the direct path from PsA to anxiety show the direct effect (total effect). Direct effect was not statistically significant. **p < 0.01, ***p < 0.001

Furthermore, pain presence was associated with both PsA and anxiety levels. Pain fully mediated the relationship between PsA and anxiety (Figure 2). The bootstrapped indirect effect was *β* = 0.61, 95% CI 0.35 *to* 0.98, *p* < 0.001. The proportion of effect mediated by pain was 0.46 (*ACME/total effect* = 0.61/1.31; 95% CI 0.25–1.15). Our sensitivity analysis found *R* ^2^ = 0.20 for residual variance and *R* ^2^ = 0.15 for total variance; the mediated effect of PsA group membership on anxiety through pain requires a relatively high correlation of *ρ* = 0.45 between residuals for the presence of the mediated effect to be invalid.

### 4.4 Prevalence of undiagnosed and untreated depression

60.1% of participants identified as being depressed using the HADS‐D cut‐off ≥8, did not have a recognized depressive disorder at the time of scan; the prevalence of unrecognized depression was only slightly smaller (58.5%) when using the more specific cut‐off ≥11. About two thirds of participants identified as depressed using the cut‐off ≥11 were not receiving any antidepressant medication at the time.

As depression is often comorbid to other psychiatric disorders, and to further eliminate any limitations of the HADS regarding specificity for major depression, we investigated presence of any psychiatric disorder in patients demonstrating depressive symptoms; 53.6% of participants with HADS‐D scores ≥11 did not have any recognized psychiatric disorder.

The prevalence of unrecognized and untreated depression was similar in the psoriasis only and the PsA group (Table 4).

**TABLE 4:** Prevalence of undiagnosed and untreated depression in patients identified as being depressed with the HADS‐D

	HADS‐D score ≥8			HADS‐D score ≥11		
	All (*n* = 178)	Psoriasis only (*n* = 123)	PsA (*n* = 55)	All (*n* = 82)	Psoriasis only (*n* = 62)	PsA (*n* = 20)
No psychiatric disorder	57.3% (102)	56.9% (70)	58.2% (32)	53.6% (44)	53.2% (33)	55.0% (11)
No depression	60.1% (107)	60.2% (74)	60.0% (33)	58.5% (48)	58.1% (36)	60.0% (12)
No antidepressant use	66.8% (119)	65.8% (81)	69.1% (38)	65.8% (54)	66.1% (41)	65.0% (13)

*Note*: All data reported as prevalence (count).

Abbreviation: HADS‐D, Hospital Anxiety and Depression scale‐Depression subscale.

### 4.5 Representativeness of sample

We investigated whether our sample was representative of the BADBIR registry as of May 2020 and made a comparison to participants who had not completed the HADS at baseline (12 558 patients; of them 3027 with PsA). Our sample was older than the rest of the registry, but there were no other sociodemographic or clinical differences between the HADS respondents and non‐respondents. However, the prevalence of depressive disorders recorded as baseline comorbidity in the PsA group was higher than in the psoriasis only group, among participants who had not completed the HADS (*p* < 0.0001), which is in contrast to the finding in our sample (HADS respondents). There were no differences in the prevalence of depression between non‐respondents and respondents for either group (Supplementary Tables S2 and S3).

## 5 DISCUSSION

Levels of depression and anxiety in psoriasis were associated with the presence of PsA, independently of other physical comorbidities. Our results replicate in part our previous and others' findings,^2^, ^16^ but also show some differences.

Compared to our previous report from a single tertiary centre, depression prevalence was overall lower in this BADBIR sample for patients with PsA (11.9% vs. 32.5% previously; HADS cut‐off ≥11), but similar among patients with psoriasis only (11.5% vs. 11.6% previously).^2^ In the present study, the association with PsA was only statistically significant when the lower HADS‐D depression cut‐off of 8 was used, but not using HADS‐D ≥11, in contrast to both our findings and those of McDonough et al.^16^ This may be explained by differences in population characteristics. BADBIR participants were recruited from dermatology centres, whereas most PsA patients in these two previous reports were recruited from rheumatology clinics. It is plausible that the BADBIR cohort has milder or currently less active PsA. PASI‐assessed psoriasis severity was higher in BADBIR, but chronic disease burden was less pronounced (15.5% of participants had tried biologics before enrolment compared to 79% of participants in our previous study). Furthermore, we here include geriatric patients (8.6% of sample) in contrast to our previous working‐age sample.^2^

A further explanation could be that the overrepresentation of depression in PsA predominantly concerns milder depression forms; or we may not have detected a true, smaller difference for severe depression due to its generally lower prevalence. This could also explain our findings in the wider cohort, where the groups differed for depression history, in contrast to our HADS‐completing sample. Although we cannot rule out effects of depression on non‐completion, the main reason for HADS missingness was the later introduction of the measure in the registry.

Finally, we cannot exclude misclassification regarding PsA in BADBIR. In our first work, patients' medical records were scrutinized by researchers, including an experienced rheumatologist, and patients without rheumatologist‐confirmed presence or absence of PsA were excluded from the analysis; we estimated that, by relying on self‐reported and dermatology nurse‐reported PsA diagnoses only, up to 9.4% participants may be misclassified through incorrect or inconclusive diagnosis.^2^ Details about PsA were not recorded in BABDIR, and it is not known whether consultant notes were cross‐examined.

In our hypothesized mediation models, pain fully mediated the effect of PsA on current depression and anxiety levels among patients with psoriasis. This raises the possibility that the overrepresentation of affective burden in PsA compared to the baseline skin disease may be attributable to the psychological and biological experience of pain. Our results align with findings in rheumatoid arthritis, where the evidence base is larger.^17^ A longitudinal study in patients with PsA found small bidirectional associations between changes in pain intensity and changes in psychological distress, after controlling for disease activity (number of swollen joint counts).^18^ Although pain has been traditionally associated with depression due to subjective psychological effects and accompanying fatigue, mobility reduction and disability, growing evidence suggests that a more complex relationship with inflammatory disease activity exists, where immune mechanisms may drive depression through pain.^19^

Furthering our understanding of the relationship between PsA, pain and depression can have a significant impact on treatment decisions and patients' quality of life. Although pain is common and debilitating in PsA, it still constitutes an unmet need in this population, only recently coming to research focus; even among patients on biologics, moderate‐to‐severe pain persists in two thirds of patients.^20^ Furthermore, depression, anxiety, and chronic pain are individual risk factors for biologic treatment discontinuation in PsA, with cumulative effects on discontinuation risk if they co‐exist.^21^ Both pain and anxiety have been associated with lower quality of life in patients with PsA.^22^ Although randomised controlled trials of antidepressants in comorbid arthritic pain and depression are scarce,^23^, ^24^ several antidepressant treatments and cognitive‐behavioural therapy have shown potential benefit for both pain and depression.^24^ For PsA patients with persistent pain and affective symptoms, an interdisciplinary management approach^25^ involving rheumatology, pain management and mental health teams may be reasonable.

The rates of undiagnosed depression in our participants with moderate‐to‐severe psoriasis (58.1%) and PsA (60.0%) were higher than those found in other populations at risk for depression, such as patients with inflammatory bowel disease (35%) and diabetes (45%), and similar to those found in primary care (52.7%).^5^, ^26^, ^27^ [Correction added on [date], after first online publication, two prevalence rates in discussion section were updated.] Interestingly, although diagnoses reported in BADBIR include those made in any medical setting, including primary care, undiagnosed depression in BADBIR was similar to the rates of depression not recognized by dermatologists in a previous report of 582 patients with psoriasis across European centres (61.4%).^4^ This further highlights how important is that dermatologists and rheumatologists screen for mood disorders in these chronic patients; as well as the need to improve relevant training and establish close partnerships with liaison psychiatric services, ideally in the form of psychodermatology clinics. Early identification and management of mental disorders in psoriasis could also prevent or significantly reduce the high psychological burden in the subpopulation with PsA, since the progression to PsA occurs on average a decade after psoriasis onset.^20^ We did not perform a similar analysis for anxiety, due to HADS‐A mainly identifying GAD and the predominantly non‐specific reporting and coding of anxiety disorders in BADBIR and non‐psychiatric settings in general.

The major strengths of this study lie in its sample size and inclusion of multiple centres from the UK and the Republic of Ireland, which ensures high external validity. To our knowledge, this is the largest study reporting an association of depression and anxiety with PsA in psoriasis, as well as the prevalence of undiagnosed and untreated depression in these populations to date.

Using BADBIR to address our study aims is subject to some limitations. BADBIR was designed as a pharmacovigilance registry for psoriasis; HADS completion and collection of other baseline data were often asynchronous; some participants may overlap with our prior single‐centre sample; and we cannot exclude some misclassification in relation to PsA as well as under‐ or overcoding of depression history depending on the data source and study centre. Furthermore, HADS does not take into account the full diagnostic criteria of major depression, importantly suicidality, concentration, sleep/appetite disturbance and feelings of worthlessness.

Our mediation analysis is subject to limitations of the cross‐sectional design, which does not allow for temporality or causality inference.^28^ We made efforts to minimize bias by using HADS scores to reflect depression levels at the time of collection and excluding pain reports dated after HADS completion. Furthermore, although we examined pain as a binary variable instead of using pain severity, adjusted for confounders and performed sensitivity analyses, we cannot exclude that depression levels may have some causal influence on the presence/absence of pain in these patients. Finally, we hypothesized pain as a mediator for this simple mediational analysis, as pain is the principal domain affecting PsA patients' quality of life according to a 13‐country study by European Alliance of Associations for Rheumatology (EULAR),^29^ is correlated with depression in PsA,^2^ and is often resistant to conventional systemic and biologic treatments.^20^, ^30^ Pain is conceptually expected to be a key component of a central mediational pathway in the PsA‐depression link possibly involving other contributing factors, such as reduced mobility, joint stiffness and fatigue. The relationships between these factors are likely complex. For example, reduced mobility may act both as a sequential mediator in the same hypothesized pathway (with pain leading to reduced mobility, leading to depression), and have additional parallel effects, driven by joint stiffness and fatigue. However, fatigue is also a symptom of depression and its role would be difficult to disentangle. We believe that findings of our exploratory analyses can help the formation of causal hypotheses in future research; however, longitudinal studies in large samples are needed to investigate these complex relationships.

## 6 CONCLUSIONS

The association of PsA comorbidity with higher anxiety in psoriasis appears to be robust and independent of confounders. The association of PsA with depression in psoriasis is replicable when milder depressive syndromes are included, but a less consistent finding for more stringent/higher‐threshold depression definitions. Our findings imply that the presence of pain may play a key role in the increased affective burden of PsA. It would be important to investigate the relationship of progression to PsA with pain, inflammatory markers and depressive syndromes in psoriasis using longitudinal cohort data. Depression remains unrecognized and untreated in a very high proportion of patients with psoriasis. It is crucial for clinicians to form trusting therapeutic alliances with these patients, establish routine mental health screening, explore and manage pain symptoms and arrange psychiatric and psychotherapeutic care where appropriate.

## AUTHOR CONTRIBUTION

**Georgia Lada**: Conceptualization (lead); Data curation (lead); Formal analysis (lead); Investigation (lead); Methodology (lead); Project administration (lead); Visualization (lead); Writing – original draft (lead); Writing – review & editing (equal). **Hector Chinoy**: Conceptualization (supporting); Funding acquisition (supporting); Methodology (supporting); Project administration (supporting); Resources (supporting); Supervision (equal); Writing – review & editing (equal). **Peter S. Talbot**: Conceptualization (supporting); Methodology (supporting); Project administration (supporting); Resources (supporting); Supervision (equal); Writing – review & editing (equal). **Richard B. Warren**: Conceptualization (supporting); Funding acquisition (supporting); Investigation (supporting); Methodology (supporting); Project administration (supporting); Resources (supporting); Supervision (equal); Writing – review & editing (equal). **C. Elise Kleyn**: Conceptualization (lead); Funding acquisition (lead); Investigation (supporting); Methodology (supporting); Project administration (lead); Resources (supporting); Supervision (lead); Writing – review & editing (equal).

## DISCLAIMER

The views expressed in this publication are those of the authors and not necessarily those of the BADBIR, NHS, the NIHR or the Department of Health.

## CONFLICT OF INTEREST

C. Elise Kleyn has received honoraria, consultant and/or research funding from Janssen, Eli Lilly, LEO, Novartis, Abbvie, UCB, Almirall, Pfizer, and L’Oréal. Hector Chinoy has received personal compensation for activities with Novartis, UCB, Lilly, Biogen, Orphazyme as a speaker, advisory board member or consultancy, grants via The University of Manchester from Novartis, UCB and MedImmune, and has received travel support from Abbvie and Janssen. Richard B. Warren has received research grants from AbbVie, Almirall, Amgen, Celgene, Janssen, Lilly, Leo, Medac, Novartis, Pfizer, and UCB and consulting fees from AbbVie, Almirall, Amgen, Arena, Astellas, Avillion, Boehringer Ingelheim, Bristol Myers Squibb, Celgene, DiCE, GSK, Janssen, Lilly, Leo, Medac, Novartis, Pfizer, Sanofi, Sun Pharma, UCB, and UNION. Georgia Lada has received speaker honoraria from Janssen, Lilly, Leo, and Novartis. Peter S. Talbot has no conflicts of interest.

## ETHICS STATEMENT

BADBIR was approved by the NHS Research Ethics Committee North West England in March 2007 (reference 07/MRE08/9); all participants gave written informed consent.

## Supporting information

Supplementary Material

## Data Availability

Restrictions apply to the availability of these data due to patient consent and licensing agreements with NHSDigital; data were used under license for this study. As such, the authors cannot make these data publicly available due to data use agreements. In order to access the data set, please see the process to apply http://www.badbir.org/Publications/.

## References

[1] Zhao SS , Miller N , Harrison N , Duffield SJ , Dey M , Goodson NJ . Systematic review of mental health comorbidities in psoriatic arthritis. Clin Rheumatol. 2020;39(1):217–25. 10.1007/s10067-019-04734-8 31486931

[2] Lada G , Chinoy H , Heal C , Warren RB , Talbot PS , Kleyn CE . Depression and suicidality in patients with psoriasis and the role of psoriatic arthritis; a cross‐sectional study in a tertiary setting. J Acad Consult Liaison Psychiatry. 2022. 10.1016/j.jaclp.2021.12.007 35017124

[3] Kimball AB , Jacobson C , Weiss S , Vreeland MG , Wu Y . The psychosocial burden of psoriasis. Am J Clin Dermatol. 2005;6:383–92. 10.2165/00128071-200506060-00005 16343026

[4] Dalgard FJ , Svensson Å , Gieler U , Tomas‐Aragones L , Lien L , Poot F , et al. Dermatologists across Europe underestimate depression and anxiety: results from 3635 dermatological consultations. Br J Dermatol. 2018;179:464–70. 10.1111/bjd.16250 29247454

[5] Li C , Ford ES , Zhao G , Ahluwalia IB , Pearson WS , Mokdad AH . Prevalence and correlates of undiagnosed depression among US adults with diabetes: the Behavioral Risk Factor Surveillance System, 2006. Diabetes Res Clin Pract. 2009;83(2):268–79. 10.1016/j.diabres.2008.11.006 19111364

[6] Goldenberg DL . The interface of pain and mood disturbances in the rheumatic diseases. Semin Arthritis Rheum. 2010;40:15–31. 10.1016/j.semarthrit.2008.11.005 19217649

[7] Burden A , Warren R , Kleyn C , McElhone K , Smith C , Reynolds N , et al. The British Association of Dermatologists’ biologic interventions register (BADBIR): design, methodology and objectives. Br J Dermatol. 2012;166(3):545–54. 10.1111/j.1365-2133.2012.10835.x 22356636

[8] Zigmond AS , Snaith RP . The hospital anxiety and depression scale. Acta Psychiatr Scand. 1983;67(6):361–70. 10.1111/j.1600-0447.1983.tb09716.x 6880820

[9] Group TE . EuroQol‐a new facility for the measurement of health‐related quality of life. Health Pol. 1990;16:199–208. 10.1016/0168-8510(90)90421-9 10109801

[10] Brennan C , Worrall‐Davies A , McMillan D , Gilbody S , House A . The Hospital Anxiety and Depression Scale: a diagnostic meta‐analysis of case‐finding ability. J Psychosom Res. 2010;69(4):371–8. 10.1016/j.jpsychores.2010.04.006 20846538

[11] Snaith RP , Zigmond AS . *The hospital anxiey and depression scale with the irritability‐depression‐anxiety scale and the leeds situational anxiety scale: manual*: Nfer‐Nelson; 1994.

[12] Madley‐Dowd P , Hughes R , Tilling K , Heron J . The proportion of missing data should not be used to guide decisions on multiple imputation. J Clin Epidemiol. 2019;110:63–73. 10.1016/j.jclinepi.2019.02.016 30878639 PMC6547017

[13] Benjamini Y , Hochberg Y . Controlling the false discovery rate: a practical and powerful approach to multiple testing. JR Statist Soc B. 1995;57(1):289–300. 10.1111/j.2517-6161.1995.tb02031.x

[14] Imai K , Keele L , Yamamoto T . Identification, inference and sensitivity analysis for causal mediation effects. Stat Sci. 2010;25(1):51–71. 10.1214/10-sts321

[15] Team RC . R: a language and environment for statistical computing; 2013.

[16] McDonough E , Ayearst R , Eder L , Chandran V , Rosen CF , Thavaneswaran A , et al. Depression and anxiety in psoriatic disease: prevalence and associated factors. J Rheumatol. 2014;41(5):887–96. jrheum. 130797. 10.3899/jrheum.130797 24692521

[17] Dickens C , McGowan L , Clark‐Carter D , Creed F . Depression in rheumatoid arthritis: a systematic review of the literature with meta‐analysis. Psychosom Med. 2002;64(1):52–60. 10.1097/00006842-200201000-00008 11818586

[18] Husted JA , Tom BD , Farewell VT , Gladman DD . Longitudinal study of the bidirectional association between pain and depressive symptoms in patients with psoriatic arthritis. Arthritis Care Res. 2012;64(5):758–65. 10.1002/acr.21602 22231988

[19] Nerurkar L , Siebert S , McInnes IB , Cavanagh J . Rheumatoid arthritis and depression: an inflammatory perspective. Lancet Psychiatr. 2019;6(2):164–73. 10.1016/s2215-0366(18)30255-4 30366684

[20] Hackett S , Ogdie A , Coates LC . Psoriatic arthritis: prospects for the future. Ther Adv Musculoskelet Dis. 2022;14. 1759720X221086710. 10.1177/1759720x221086710 PMC8966104 35368374

[21] Katz G , Ogdie A , Baker JF , George MD . Association between depression, anxiety, chronic pain, or opioid use and tumor necrosis factor inhibitor persistence in inflammatory arthritis. Clin Rheumatol. 2022;41(5):1–9. 10.1007/s10067-021-06045-3 35084601 PMC9058194

[22] Kotsis K , Voulgari PV , Tsifetaki N , Machado MO , Carvalho AF , Creed F , et al. Anxiety and depressive symptoms and illness perceptions in psoriatic arthritis and associations with physical health‐related quality of life. Arthritis Care Res. 2012;64(10):1593–601. 10.1002/acr.21725 22556134

[23] Richards BL , Whittle SL , Buchbinder R . Antidepressants for pain management in rheumatoid arthritis. Cochrane Database Syst Rev. 2011. 10.1002/14651858.CD008920.pub2 PMC12214451 22071859

[24] IsHak WW , Wen RY , Naghdechi L , Vanle B , Dang J , Knosp M , et al. Pain and depression: a systematic review. Harv Rev Psychiatry. 2018;26(6):352–63. 10.1097/hrp.0000000000000198 30407234

[25] Geenen R , Overman CL , Christensen R , Asenlof P , Capela S , Huisinga KL , et al. EULAR recommendations for the health professional’s approach to pain management in inflammatory arthritis and osteoarthritis. Ann Rheum Dis. 2018;77:797–807. 10.1136/annrheumdis-2017-212662 29724726

[26] Lewis K , Marrie RA , Bernstein CN , Graff LA , Patten SB , Sareen J , et al. The prevalence and risk factors of undiagnosed depression and anxiety disorders among patients with inflammatory bowel disease. Inflamm Bowel Dis. 2019;25(10):1674–80. 10.1093/ibd/izz045 30888037

[27] Mitchell AJ , Vaze A , Rao S . Clinical diagnosis of depression in primary care: a meta‐analysis. Lancet. 2009;374(9690):609–19. 10.1016/s0140-6736(09)60879-5 19640579

[28] Fiedler K , Schott M , Meiser T . What mediation analysis can (not) do. J Exp Soc Psychol. 2011;47:1231–6. 10.1016/j.jesp.2011.05.007

[29] Gossec L , de Wit M , Kiltz U , Braun J , Kalyoncu U , Scrivo R , et al. A patient‐derived and patient‐reported outcome measure for assessing psoriatic arthritis: elaboration and preliminary validation of the Psoriatic Arthritis Impact of Disease (PsAID) questionnaire, a 13‐country EULAR initiative. Ann Rheum Dis. 2014;73(6):1012–9. 10.1136/annrheumdis-2014-205207 24790067

[30] Conaghan P , Strand V , Alten R , Sullivan E , Blackburn S , Huneault L , et al. OP0107 Pain still remains a high unmet need among psoriatic arthritis patients receiving existing biologic treatment: results from a multi national real‐world survey. Abstract: OP0107. Ann Rheum Dis. 2017;76:96–7.27165179

[31] Finlay AY , Khan G . Dermatology Life Quality Index (DLQI)—a simple practical measure for routine clinical use. Clin Exp Dermatol. 1994;19(3):210–6. 10.1111/j.1365-2230.1994.tb01167.x 8033378

