# Supplementary material for: Associations between psoriatic arthritis and mental health among patients with psoriasis: A replication and extension study using the British Association of Dermatologists Biologics and Immunomodulators Register (BADBIR)
Source: Skin Health Dis. 2022 Jul 6;2(4):e149. doi: 10.1002/ski2.149 (PMC9720192; doi:10.1002/ski2.149)
Supplement: Supplementary file 1 — Supplementary Material [file SKI2-2-e149-s001.docx]

**Supplementary Information**

**Appendix 1**

**Data source**

BADBIR is a large prospective cohort study ongoing since September 2017 in over 160 sites in the United Kingdom and the Republic of Ireland. Its main objective is to serve as a pharmacovigilance registry of patients with moderate-to-severe psoriasis. BADBIR includes three treatment-based cohorts of patients and compares a cohort receiving biologic therapies with two further cohorts treated with conventional systemic and oral small-molecule therapies. Patient data are collected at enrolment and in regular follow-ups. BADBIR was approved by the NHS Research Ethics Committee North West England in March 2007 (reference 07/MRE08/9); all participants gave written informed consent.

**Criteria for exclusion of PASI scores used in the analysis**

We excluded PASI scores where: a) PASI was dated outside a 4-month window of the Hospital Anxiety and Depression Scale (HADS) assessment; b) PASI was performed more than 2 weeks before the HADS assessment in patients where psoriasis treatment was initiated following or on the day of the PASI assessment; c) PASI scores were performed more than 2 weeks before the HADS assessment and less than 6 weeks following initiation of psoriasis treatment; d) HADS was administered more than 2 weeks before the PASI and psoriasis treatment was initiated following or on the day of the HADS assessment; e) HADS was administered more than 2 weeks before the PASI and less than 6 weeks following initiation of psoriasis treatment. The 2-week time interval was chosen to reflect the time criterion for major depressive disorder and the 6-week interval to reflect the onset of action window for most biologics ^1^. If more than one PASI baseline assessments were reported for a participant, we selected the PASI score performed on the date closest to the HADS assessment.

**Representativeness of our sample compared to the rest of BADBIR**

We investigated whether our sample was representative of the BADBIR registry as of May 2020 and compared it to those participants who had not completed the HADS at baseline. Due to the vast majority of participants completing the HADS being in the biologics cohort, we selected the information from the biologics cohort for analysis, in cases where participants without HADS had been registered in more than one cohort since their consent.

The cohort who completed the HADS was adequately representative of the BADBIR. Participants who completed the HADS were slightly but significantly older at consent, but did not differ for gender, disease onset, ethnicity or BMI with the rest of the cohort. The proportions of people reporting pain in the subsample with HADS were lower when examining the samples as a whole and also when examining patients with psoriasis only and patients with PsA separately (p<0.001).

We did not compare for employment status due to its relatively high missingness in the rest of the cohort. As many participants had multiple PASI scores at baseline and PASI scores were filtered in our analysis based on their time relation to the HADS administration, selecting matched PASI values for comparison was impossible.

**Table S1: Overall HADS scores for depression and anxiety in patients with and without psoriatic arthritis (PsA)**

| **HADS total scores** | **Crude scores** | | | **Adjusted model ^a^** | |
| --- | --- | --- | --- | --- | --- |
|  | **Median (IQR)** | | ***p*-value ^b^** | ***β* adjusted (95% Confidence Intervals)** | ***p-*value** |
|  | **Psoriasis only** | **PsA** |  |  |  |
| **Depression subscale** | 3.0 (6.0) | 5.0 (6.5) | **0.012** ^c^ | 0.84 (0.10, 1.57) | **0.037** ^d^ |
| **Anxiety subscale** | 5.0  (7.0) | 7.0 (7.5) | **0.012** ^e^ | 1.32 (0.54, 2.11) | **0.012** ^f^ |
| Notes: HADS=Hospital Anxiety and Depression Scale. ^a^ adjusted for age, gender, ethnicity, presence of physical comorbidities, psoriasis severity and body mass index. ^b^ Mann Whitney U test ^c^ BH (Benjamini-Hochberg)-corrected; original p-value=0.003 ^d^ BH-corrected; original p-value 0.025 ^e^ BH-corrected; original p-value 0.002 ^f^ BH-corrected; original p-value < 0.001 | | | | | |

| **Table S2: Comparison of baseline characteristics between our sample and BADBIR participants who have not completed the Hospital Anxiety and Depression Scale (HADS) at baseline** | | | | | | | | | | |
| --- | --- | --- | --- | --- | --- | --- | --- | --- | --- | --- |
|  | **All BADBIR** | | | **HADS not available** | | | **HADS available** | | | **p-value ^a^** |
|  | **All** | **Ps** | **PsA** | **All** | **Ps** | **PsA** | **All** | **Ps** | **PsA** |  |
| **Group** | 13265 | 76% (10075) | 24% (3190) | 12558 | 76% (9531) | 24% (3027) | 707 | 76% (540) | 24% (167) | 0.555 |
| **Cohort; biologics** | 99.9% (13258) | 99.9% (10068) | 100.0% (3190) | 99.9% (12552) | 99.9% (9525) | 100.0% (3027) | 99.0% (700) | 99.3% (536) | 98.2% (164) | **0.016** |
| **Gender; female** | 41% (5471) | 41% (4009) | 46% (1462) | 41% (5166) | 40% (3778) | 46% (1388) | 43% (305) | 42% (229) | 45% (76) | 0.311 |
| **Age at consent in yrs, mean (sd)** | 45.1 (13.4) | 44.3 (13.5) | 47.6 (12.5) | 44.9 (13.4) | 44.2 (13.5) | 47.5 (12.5) | 46.7 (13.0) | 45.9 (13.1) | 49.4 (12.4) | **<0.001** |
| **Ethnicity; white** | 90% (11952) | 90% (9120) | 89% (2832) | 90% (11322) | 90% (8626) | 89% (2696) | 89% (630) | 91% (490) | 84% (140) | 0.156 |
| **Psoriasis age at onset in yrs, median (IQR)** | 21.0 (18.0) | 21.0 (17.0) | 22.0 (18.0) | 21.0 (17.0) | 20.0 (17.0) | 22.0 (17.0) | 22.0 (18.0) | 22.0 (18.0) | 23.0 (19.2) | 0.070 |
| **PsA age at onset in yrs, median (IQR)** |  |  | 39.0 (18.0) |  |  | 39.0 (18.0) |  |  | 38.0 (16.7) | 0.404 |
| **BMI** | 30.0 (8.8) | 29.7 (8.9) | 30.7 (8.6) | 29.9 (8.8) | 29.7 (8.9) | 30.7 (8.5) | 30.3 (9.1) | 30.1 (8.3) | 31.6 (9.5) | 0.052 |
| **Pain (yes)** | 59.5% (6995) | 53.5% (4778) | 78.5% (2217) | 59.9% (6628) | 53.9% (4526) | 78.9% (2102) | 53.6% (367) | 47.5%  (250) | 71.7 %  (117) | **<0.001** |
| Notes: ^a^ all available vs all unavailable. Ps=psoriasis only; PsA=psoriatic arthritis; IQR=interquartile range; sd=standard deviation; BMI=Body Mass Index. P-values <0.05 are reported in bold. | | | | | | | | | | |

| **Table S3: Prevalence of reported depressive disorders in our sample and BADBIR participants who have not completed the Hospital Anxiety and Depression Scale (HADS) at baseline** | | | | |
| --- | --- | --- | --- | --- |
| **Cohort** | **Group** | **N** | **Depressive Disorders prevalence % (count)** | **p-value**  **(Psoriasis only vs Psoriatic arthritis)** |
| **All BADBIR** | **All** | 13,265 | 22.5% (2992) |  |
|  | **Ps** | 10,075 | 21.0% (2119) |  |
|  | **PsA** | 3,190 | 27.4% (873) |  |
|  | **p-value** |  |  | **<0.0001** |
| **HADS not available** | **All** | 12,558 | 22.6% (2845) |  |
|  | **Ps** | 9,531 | 21.1% (2009) |  |
|  | **PsA** | 3,027 | 27.6% (836) |  |
|  | **p-value** |  |  | **<0.0001** |
| **HADS available** | **All** | 707 | 20.8% (147) |  |
|  | **Ps** | 540 | 20% (108) |  |
|  | **PsA** | 167 | 23% (39) |  |
|  | **p-value** |  |  | 0.409 |
| Note: P-values <0.05 are reported in bold. | | | | |

**References**

1 Papp KA, Lebwohl M. Onset of Action of Biologics in Patients With Moderate-to-Severe Psoriasis. J Drugs Dermatol 2017; **17**: 247-50.
